# Proof-of-concept study linking ex vivo sensitivity testing to neoadjuvant anthracycline-based chemotherapy response in breast cancer patients

**DOI:** 10.1038/s41523-023-00583-6

**Published:** 2023-09-30

**Authors:** Marjolijn M. Ladan, Titia G. Meijer, Nicole S. Verkaik, Cecile de Monye, Linetta B. Koppert, Esther Oomen-de Hoop, Carolien H. M. van Deurzen, Roland Kanaar, Julie Nonnekens, Dik C. van Gent, Agnes Jager

**Affiliations:** 1 Department of Molecular Genetics, Erasmus MC Cancer Institute, University Medical Center Rotterdam, Rotterdam, The Netherlands; 2 Oncode Institute, Rotterdam, The Netherlands; 3 Department of Radiology & Nuclear Medicine, Erasmus MC Cancer Institute, University Medical Center Rotterdam, Rotterdam, The Netherlands; 4 Department of Surgery, Erasmus MC Cancer Institute, University Medical Center Rotterdam, Rotterdam, The Netherlands; 5 Department of Medical Oncology, Erasmus MC Cancer Institute, University Medical Center Rotterdam, Rotterdam, The Netherlands; 6 Department of Pathology, Erasmus MC Cancer Institute, University Medical Center Rotterdam, Rotterdam, The Netherlands

**Keywords:** Breast cancer, Tumour biomarkers

## Abstract

We developed a functional ex vivo anthracycline-based sensitivity test. Surgical resection material of primary breast cancer (BC) was used to determine criteria for the ex vivo sensitivity assay based on morphology, proliferation and apoptosis. Subsequently, a proof-of-concept study was performed correlating results of this assay on primary BC biopsies with in vivo response after treatment with anthracycline-containing neoadjuvant chemotherapy (NAC). Cut off values for the ex vivo anthracycline-based sensitivity test were established based on analysis of 21 primary breast tumor samples obtained after surgery. In the proof-of-concept study based on a new set of tumor biopsies, 41 patients were included. Eight biopsies did not contain tumor cells and three patients could not be biopsied for various reasons. In the remaining 30 biopsies, the success rate of the ex vivo test was 77% (23/30); six out of seven failed tests were due to excessive apoptosis, our pre-specified test criteria. Of the 23 patients with a successful ex vivo test result, three patients did not undergo NAC after the biopsy. Here we report the ex vivo anthracycline-based sensitivity assay is feasible on biopsy material and shows 75% concordance between ex vivo outcomes and in vivo MRI response. Unfortunately, the percentage of unsuccessful tests is rather high. This study provides the foundation for further development of ex vivo sensitivity assays.

## Introduction

The prognosis of primary breast cancer (BC) has improved over the past decades, largely due to better locoregional and especially systemic treatment options^1,2^, but at the cost of overtreatment. Approximately 30–40% of patients whose disease would recur after surgery can now be cured by pre- or post-operative chemotherapy. Unfortunately, this also means that 60–70% of these patients will develop recurrent disease in spite of the chemotherapy. Despite the encouraging developments of targeted therapies, chemotherapy is expected to remain the cornerstone of BC treatment for decades to come. It is therefore essential to develop biomarkers which assess the sensitivity of breast tumors to chemotherapy, preferably before starting the treatment.

The search for predictive biomarkers for response to chemotherapy has predominantly focused on alterations in its main target. The most extensively studied example for BC is anthracycline sensitivity of tumors and the genomic alterations in those tumors to the main anthracycline target, topoisomerase 2-alpha (TOP2A). For example, patients with tumors harboring TOP2A aberrations benefitted more from anthracycline-based therapy than patients with tumors without TOP2A aberrations in an individual patient-level pooled analysis from five adjuvant phase III trials comparing anthracycline-based therapies with combination therapy of cyclophosphamide, methotrexate and fluorouracil^3–5^. However, the efficacy of anthracycline has also been demonstrated in patients with tumors without TOP2A abnormalities. Desmedt et al. focused on developing the so-called A-Score, a combination of three gene expression profiles with a strong negative predictive value, for selecting anthracycline-resistant tumors^6^. Although its predictive value was confirmed using three other retrospective studies, the results of these studies were less clear. Moreover, prospective validation is lacking and the test itself is still only performed on frozen tumor material.

An alternative approach to predict sensitivity is direct analysis of tumor response to chemotherapy. Recently, we developed a technology to maintain viability, tissue integrity and proliferation of BC tissue slices ex vivo for up to 1 week^7–9^, keeping cells in their natural (micro)environment. This allows testing of tissue responses to various types of treatment^9^. With this technique we previously showed that outcome parameters could be developed for the response of these human BC slices to anthracycline-based chemotherapy, platinum salts and taxanes^8,10^. A correlation of these ex vivo sensitivity assays with the in vivo response, however, was still lacking.

In the current study we further optimized the ex vivo sensitivity assay for anthracycline-based chemotherapy and performed a clinical proof-of-concept study for the correlation with the in vivo response. The BREAST study correlates this ex vivo sensitivity assay on tumor biopsies with the in vivo tumor response by breast MRI after treating these patients with anthracycline-containing NAC. The main goal was to determine whether this sensitivity assay was suitable for further validation or whether structural adjustments were needed before further validation.

## Results

### Part I: Optimizing the ex-vivo anthracycline-based sensitivity assay

**Fig. 1:**
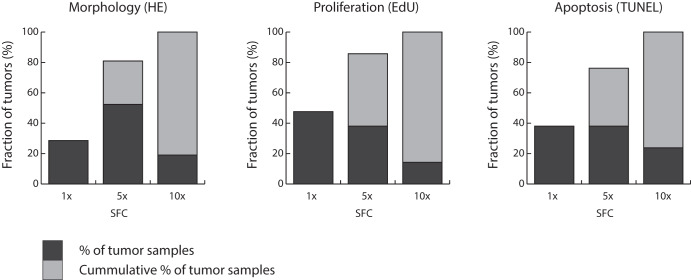
Morphology, proliferation and apoptosis after FAC treatment. Results of 21 tumors analyzed for morphology (HE), proliferation (EdU) and apoptosis (TUNEL). Percentage of tumors showing effect of the treatment and cumulative percentages are given. The *X*-axis shows the concentrations of FAC relative to the standard FAC concentration (SFC), *Y*-axis shows the percentage of all tumors reacting at this concentration.

We first developed a scoring system for ex vivo anthracycline-based sensitivity testing. We determined morphology, proliferation and apoptosis after incubation for 3 days with various concentrations of anthracycline-containing chemotherapy for 21 primary BC samples. In the primary setting, a total of two samples did not lead to a successful test. The three concentrations depicted in Fig. 1 appeared to be informative. Based on the morphology, proliferation and apoptosis results of these 21 evaluable samples, we propose criteria for the degree of anthracycline sensitivity (Table 1).

**Table 1:** Chosen cut off values for the ex vivo sensitivity test based on three separate components.

Overall ex vivo test result	Separate component of the ex vivo sensitivity test		
	EdU	TUNEL	HE
Resistant	Still proliferative cells in 5× SFC	<40% apoptosis in 5× SFC	Mostly intact cells in 5× SFC
Intermediate sensitive	Still proliferative cells in 1× SFC No proliferative cells in 5× SFC	<40% apoptosis in 1× SFC >40% apoptosis in 5× SFC	Mostly intact cells in 1× SFC Mostly dead cells in 5× SFC
Sensitive	No proliferative cells in 1× SFC	>40% apoptosis in 1× SFC	Mostly dead cells in 1× SFC

Proliferation was measured by EdU, apoptosis by TUNEL and morphology by HE. SFC standard FAC concentration, i.e., 23 µM F (5-Fluorouracil), 0.5 µM A (doxorubicine) and 11 µM C (4-hydroperoxy cyclophosphamide).

To establish the criteria for predicting ex vivo response, we took into account the limited availability of tumor material in biopsies. Therefore, two concentrations were chosen for ex vivo anthracycline treatment. Some tumors did not respond to the 5× SFC, while other tumors already responded to 1× SFC. This resulted in criteria to select anthracycline-resistant tumors, shown in Table 1. We decided to use the 5× SFC to identify resistant tumors, because resistance is rare and there were few tumors which were not affected by this concentration only. Furthermore, one of these tumors (M072) had been found to be anthracycline-resistant upon neoadjuvant treatment in vivo. On the other hand, the 1× SFC concentration defined a group of sensitive tumors. According to these criteria, the ex vivo sensitivity test results of these tumors were classified as resistant, intermediate sensitive and sensitive for each assay and tumors were classified according to the average sensitivity level of the three assays.

### Part II: The BREAST study

**Fig. 2:**
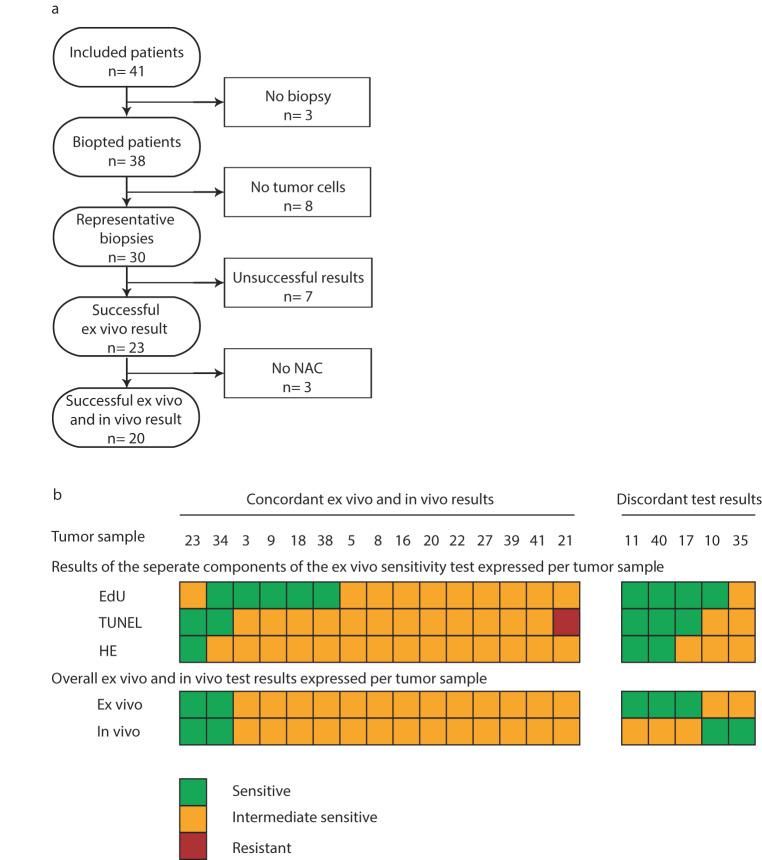
Overview of the BREAST study results. **a** Number of patients included and reason for exclusion in analysis. **b** Ex vivo sensitivity test: proliferation was measured by EdU/keratin, apoptosis by TUNEL and morphology by HE together resulting in an overall final ex vivo test result per tumor sample based on the criteria summarized in Table 1. In vivo response was measured by MRI response after four courses of anthracycline-containing chemotherapy and classified by RECIST 1.1.

After defining these starting parameters on 21 primary resected tumors, we took the next step toward clinical validation by comparing the ex vivo sensitivity to in vivo tumor responses. A total of 41 new BC patients signed informed consent for the BREAST study. Three patients ultimately did not undergo a biopsy. The lack of tumor cells in eight biopsies left 30 patients with an adequate tumor biopsy (Fig. 2a).

### Ex vivo test results

The success rate of the ex vivo sensitivity assay was 77% (23 out of 30 samples). Infection during culturing in one of the samples prevented an interpretable ex vivo test. The other six failed tests were due to the (pre-specified) test criterion that the fraction of apoptotic cells should not exceed 40% in the untreated slice. We did not find any significant differences in tumor characteristics between successful and unsuccessful test results (Table 2). Of the 23 evaluable ex vivo tests, anthracycline-sensitivity was demonstrated in six patients (26%; 6/23) and intermediate sensitivity in 17 patients (74%; 17/23) (Supplementary Fig. 1). No resistant tumors were found.

**Table 2:** Tumor characteristics for successful and unsuccessful ex vivo sensitivity test results.

	Successful ex vivo test (*n* = 23)	Unsuccessful ex vivo test (*n* = 7)	*P* value
Receptor status^a^			0.61
• ER + , HER2−	11 (48%)	3 (43%)	
• ER−, HER2−	8 (35%)	4 (47%)	
• ER + , HER2+	4 (17%)	0 (0%)	
Bloom & Richardson grade^b^			0.66
• Grade 2	16 (70%)	4 (57%)	
• Grade 3	7 (30%)	3 (43%)	
Proliferation (% Ki67)			0.34
• <10%	6 (26%)	3 (43%)	
• >10%	17 (74%)	3 (43%)	
• Not present	0	1 (14%)	
In vivo MRI response			1.00
• Sensitive	4 (18%)	1 (12%)	
• Intermediate	17 (77%)	6 (75%)	
• Resistant	0	0	

Unsuccessful tests were tests where an ex vivo test result could not be given due to the lack of one or more individual test results. Two patients were not included in the in vivo results since they did not receive NAC treatment.

^a^No tumors with ER−, HER2+ receptor status.

^b^No grade 1 tumors. ER estrogen receptor, HER2 Her2-neu receptor. One patient was ER-PR + HER2− and was classified as TN. *P* value was measured by a two-sided Fisher exact test.

### In vivo test results

Of the 23 patients with a successful ex vivo test result, three patients ultimately did not undergo NAC before resection. The in vivo tumor responses based on MRI response after four courses of anthracycline-containing chemotherapy showed sensitivity ( > 50% decrease in tumor size) to anthracyclines in four patients (20%) and intermediate sensitivity ( < 50% decrease and <20% increase in tumor size) to anthracyclines in 16 patients (80%). No tumor showed anthracycline resistance.

In our proof-of-concept study, the ex vivo sensitivity assay and the in vivo tumor response was concordant in 75% (15/20; CI: 53-89%) and discordant in 25% of the cases (5/20, CI: 11–47%; Fig. 2b). In three out of five discordant samples, the assay predicted a sensitive response while MRI response was intermediate sensitive, and in two samples the ex vivo prediction was intermediate sensitive while MRI response showed sensitive results. Compared to the discordant tumors, the concordant tumors were more often grade 3 and had a higher Ki67 score (Table 3), although these differences did not reach statistical significance.

**Table 3:** Tumor characteristics of the tumors with concordant in vivo and ex vivo sensitivity test results and of tumors with discordant in vivo and ex vivo sensitivity test results.

	Concordant (*n* = 15)	Discordant (*n* = 5)	*P* value
Receptor status^a^			0.21
• ER+, HER2−	5 (33%)	4 (80%)	
• ER−, HER2−	6 (40%)	(10%)	
• ER+, HER2+	4 (27%)	0	
Bloom & Richardson grade^b^			0.26
• Grade 2	9 (60%)	(100%)	
• Grade 3	6 (40%)	0	
Ki67			0.07
• <10%	2 (13%)	3 (60%)	
• >10%	13 (87%)	2 (40%)	

^a^No tumors with ER−, HER2+ receptor status.

^b^No grade 1 tumors. ER estrogen receptor, HER2 Her2-neu receptor. *P* value was measured by a two-sided Fisher exact test.

**Fig. 3:**
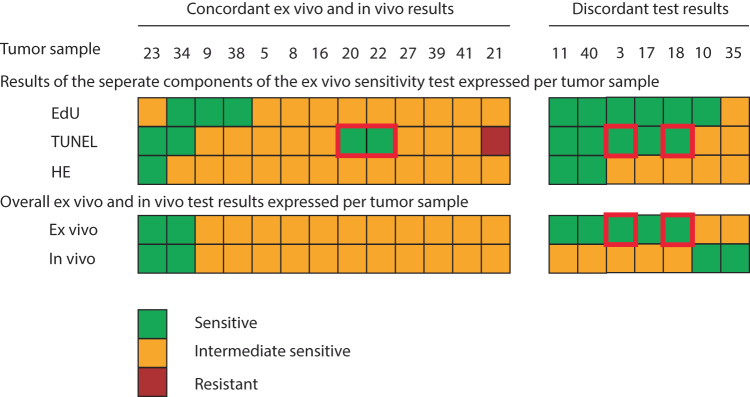
Concordance between ex vivo sensitivity test and in vivo MRI outcome based on an alternative TUNEL cut off. TUNEL cut off values were changed to an increase of 20% relative to the untreated slice (instead of a fixed level of 40%). Further ex vivo sensitivity test were the same as in Fig. 2, together resulting in an overall final ex vivo test result per tumor sample based on the criteria mentioned in Table 1. In vivo response was evaluated by MRI after four courses of anthracycline-containing chemotherapy and classified by RECIST 1.1. The samples which changed sensitivity when the cut off criteria changed from 40% to an increase of 20% are highlighted in red boxes. Two tumors changed in overall ex vivo outcome and were now discordant (samples 3 and 18).

Pre-planned analyses were included in the protocol to explore whether different cut-off values for the three parameters of the ex vivo sensitivity test would lead to a higher degree of concordance. A different definition of TUNEL staining to determine sensitivity, i.e., an absolute increase of >20% TUNEL positivity instead of a fixed cut-off ( > 40%) in response to anthracycline was explored. This classification might have the advantage that it can take into account the potentially large variation in TUNEL positivity of the untreated slice between biopsies of individual tumors, which might result in a better classification of tumors with relatively high apoptotic levels in untreated samples. We evaluated a threshold of 20% increase of TUNEL positive DAPI pixels relative to the untreated slice. This resulted in two tumors (3 and 18) going from an intermediate sensitive to a sensitive ex vivo test result (Fig. 3 and supplementary Fig. 2). However, these two tumors showed an intermediate sensitive in vivo response and thus an overall decrease in concordance from 75% (15/20) to 65% (13/20). Therefore, we rejected this alternative scoring system. We also evaluated less stringent criteria for apoptosis. Using <50% TUNEL positivity in the untreated tumor slice as mandatory criteria resulted in three additional successful tests (success rate 26/30; 87%), while <60% TUNEL positivity yielded four additional successful tests (success rate 27/30; 90%). However, this altered threshold (to 50% or 60%) would result in changes in our criteria (to higher than 50% or higher than 60%), resulting in more misclassified samples. Therefore, we conclude that there was no benefit in changing our initial scoring parameters.

## Discussion

In this study, we describe an ex vivo anthracycline-based sensitivity test that is feasible on biopsies, which showed high concordance with the in vivo anthracycline-sensitivity response (75%). Further improvement of the rate of concordance by adapting thresholds for outcome parameters could not be achieved. The number of unsuccessful ex vivo test results was unfortunately rather high, mainly due to high levels of cell death in the tumor samples. This study is a starting point for further development of predictive ex vivo chemotherapy sensitivity tests and identifies several aspects that require further improvements.

In our proof-of-concept study, as many as 41 patients were needed to select 20 suitable patients. Six patients (6/41 = 15%) dropped out due to logistical reasons (biopsy in three patients could not be scheduled before starting NAC; treatment plan of three patients changed to upfront breast surgery). Unexpectedly, eight out of 38 (21%) biopsies did not contain tumor cells and therefore these patients could not be included in our analysis. This is a high percentage and might be explained by the fact that the biopsies for this study were taken after previous biopsies to establish the primary diagnosis and mostly at exactly the same location. The previous biopsies may have caused local changes in the ultrasound image (e.g., a hematoma), increasing the likelihood of sampling errors.

Of the 30 biopsies which could undergo the ex vivo sensitivity test, seven biopsies (23%) were found to yield an unsuccessful test result. Six of the seven test results (86%) were unsuccessful due to the predefined test criteria, and thus only one biopsy was unsuccessful due to the failure of the test itself. The predefined criteria for scoring the ex vivo assay were a sufficient number of proliferating tumor cells and less than 40% apoptotic cells in the biopsy material. This was not a problem for the surgical tissue sections (21 of 23 tumors), but proved to be much more challenging in the relatively small breast tumor biopsies. Increasing the apoptosis limit to <50% in the pre-treatment biopsy sample did not improve the outcome. We therefore propose to start from at least two biopsies in follow-up studies, one in the center and one at the periphery of the tumor.

Five out of 20 tumors showed discordant results, either overestimating or underestimating tumor sensitivity. This meets our predefined requirement of at least 55% concordance and is therefore sufficiently hopeful to warrant further investigation of the ex vivo assay.

We considered several issues that may negatively influence the degree of concordance between ex vivo and in vivo outcome, which can be partially avoided. First, tumor heterogeneity may hamper correct prediction and could be responsible for both under- and overestimation of the ex vivo sensitivity test results. Previously mentioned two biopsies at different positions could better probe this heterogeneity. Second, differences in drug accessibility of the tumor can be caused by the vasculature^11,12^; this would influence the concentration of the chemotherapeutic compounds in vivo, whereas the ex vivo concentration is only dependent on diffusion into the tumor slice. Insufficient vascularization in vivo would therefore mainly result in overestimation of the tumor sensitivity ex vivo. Including vascular markers in the analysis could provide more insight into this. Third, systemic differences, caused by differences of various organs, influence the in vivo response. For example, detoxification or metabolic activation in the liver or secretion via urine or faeces is not mimicked in the ex vivo assay and could lead to both under- and overestimation of the ex vivo sensitivity test result. Fourth, the ex vivo tissue slice system has not been optimized for immune components, such as tumor-infiltrating leukocytes (TILs), which are an important factor in determining chemotherapy sensitivity^13^. Although TILs can be present in tumor slices, changes occur during treatment in the patient^14^, which is not mimicked faithfully in our ex vivo system. It is interesting that concordance is high (75%) in spite of all these possible factors that could cause differences between ex vivo and in vivo responses, which probably means that intrinsic tumor cell sensitivity is the overruling parameter determining therapy response in the majority of patients.

Two limitations should be mentioned. First, the ex vivo test was set up with a FAC regimen, because this was the standard of care when the test was developed. It is possible that this is significantly different from an AC sensitivity assay. However, as the effect of 5-Fluorouracil (F) in addition to AC is very limited according to the literature^15^, it is not likely that this would change the conclusions significantly. Second, pathological response could not be used in this study because the anthracycline-based therapy was directly followed by taxane treatment, before surgical removal of the tumor. A study performed on MRI size prediction, in comparison to pathological measurement, stated a wrong size prediction in 28% of the cases. Of these 28%, tumor size was overestimated in 93%. This ensures us that response to FAC therapy would more likely be underestimated instead of overestimated^16^.

An interesting observation is that simplification of the ex vivo test seems possible. Based on our results shown in Fig. 2, one could argue that apoptosis alone can be a good predictor of sensitivity without the addition of morphology and proliferation, as those markers influence concordance for only one sample. This would increase the applicability of the ex vivo assay in clinical practice, as TUNEL staining can be scored automatically, while HE must be analyzed by a trained eye and the EdU positive cells need to be counted manually, which is prone for inter-observer variability. However, the presence of proliferation and tumor will always have to be determined on the untreated slice.

Recently, we have taken the first steps in developing ex vivo sensitivity assays for taxanes and platinum derivates, two chemotherapeutic agents which are frequently used in BC treatment^10^. Since both taxanes and anthracyclines are often used in the (neo)adjuvant setting in BC treatment, it might be of interest to use both ex vivo sensitivity assays simultaneously on different slices of the same biopsy to explore the possibility to optimize treatment by selecting the most optimal treatment for each patient. However, the limited availability of tumor material is the limiting factor. In the future, culture conditions could be optimized using a Cancer-on-Chip approach, that we recently developed^17^. This would require a minimal amount of tumor material; we estimate that two biopsies should be enough to test several conditions. Cancer-on-Chip could also be combined with direct microscopic imaging and/or analysis of outflow media to maximize the amount of information extracted from a single experiment. The assay conditions determined in this study provide a starting point for such a multiplexed chemotherapy sensitivity test.

In conclusion, in this study we showed that the ex vivo anthracycline sensitivity assay can be performed on small biopsies and that the concordance with in vivo outcome is high. Further development should focus on not only expanding the number of patients to confirm the presented data but also on further developing other ex vivo chemotherapeutic sensitivity tests. Using multiple different drugs in one assay would have the ultimate goal of predicting which chemotherapeutic agents have the highest potential for efficacy at the individual patient level.

## Methods

### Part I: Development of the ex vivo anthracycline-based sensitivity assay

A set of previously published tumors was combined with new samples^8^. After surgical removal of the primary BC, viable tumors were sent to the pathology department. Samples from breast tumors, where sufficient tumor material was present, were donated for our study, approved by the Erasmus MC Medical Ethical Committee (MEC-2011-098). Samples were collected in customized breast tissue medium^7^, sliced into 300 µm slices using the Leica VT 1200 s Vibratome, and cultured as described^7,8^. Briefly, slices were cultured on an orbital shaker (60 rpm) in a 5% CO_2_ and 37°C atmosphere. Slices were treated with 5-fluorouracil (F), doxorubicin (A) and 4-hydroperoxy cyclophosphamide (C) at various concentrations for 3 days. The concentrations differed, but the ratio (46:1:22) remained the same between the three chemotherapeutics and was based on the clinically used ratio^18^. Concentration 1× is 23 µM F, 0.5 µM A and 11 µM C and was called the standard FAC concentration (SFC). This combination of chemotherapeutic agents was the standard treatment in daily practice at the time of test development. 5-Ethynyl-2′-deoxyuridine (EdU, 30 µM) was added 2 h before formalin fixation as a marker for proliferation. After paraffin embedding, 4 µm sections were used for staining. The outcome of the ex vivo assay was determined by evaluation of three parameters; histology (HE-staining and keratin staining), proliferation (EdU staining combined with the keratin staining to identify tumor cells) and apoptosis (TUNEL staining)^8^. The first 14 primary BC samples have been described in detail elsewhere^8^. With the results from both the previously published samples and the newly added samples, criteria have been established with which the proof-of-concept study was performed.

### Part II: Proof-of-concept study: the BREAST study

All BC patients who had been scheduled to start with anthracycline-containing NAC without concomitant taxane treatment and had WHO performance status 0 or 1 without any psychologically interfering problems were potentially eligible for inclusion in the study. After written informed consent, a pre-treatment fresh tumor biopsy (14 Gauge) of the breast was collected in customized breast tissue medium^7^. The ex vivo response per tumor was obtained by performing the ex vivo anthracycline-based chemotherapy sensitivity assay developed in part I on at least three fresh 300 µm tumor slices; untreated and at least two different FAC concentrations. Before the sensitivity assay could be performed, the untreated tumor slices had to contain (1) tumor cells, proven by HE and keratin staining, (2) EdU positive cells in the tumor area and (3) <40% TUNEL positive DAPI pixels. Three subgroups were defined based on morphology, proliferation and apoptosis; sensitive, intermediate sensitive and resistant. The in vivo response was evaluated from contrast enhanced breast MRI before starting NAC and after four courses of anthracycline-containing treatment. In vivo response was defined as complete response, partial response, stable disease and progressive disease using the Response Evaluation Criteria in Solid Tumors (RECIST) 1.1. on MRI^19^. The ex vivo response was considered concordant when one of the following three results was obtained: a sensitive response was coupled to an in vivo response of >50% decrease in tumor size (Table 1), an intermediate sensitive ex vivo response was coupled to an intermediate sensitive MRI response ( < 50% decrease and <20% increase) or a resistant response was taken as concordant when progressive disease ( > 20% increase) was detected on the MRI scan. This trial was approved by the medical ethics commission of the Erasmus Medical Centre (MEC16-600) and was admitted to the Netherlands Trial Register (NL 5588).

Results of the ex vivo anthracycline sensitivity test can be known between 7 and 14 days.

### Statistical analysis

For this pilot, we aimed to find a concordance between the ex vivo and in vivo response of at least 55% (P1), which was considered sufficiently promising to warrant further research. If concordance was <25% (P0) the ex vivo assay in its current format has no advantage over patient selection according to present standards. For the sample size calculation, the optimal Simon 2-stage design was applied^20^. With an α and β of both 0.10, the required number of patients with a successful ex vivo test result to be included was 20. Of these 20 patients, 55% needed to be concordant to draw a final conclusion. One interim analysis was planned after the results from the first nine evaluable patients were available. If ≤2 patients had a concordant result or >5 patients had tumors classified resistant by the ex vivo anthracycline-based sensitivity assay showing a good in vivo response, the study would have been terminated prematurely. Only patients with a representative biopsy and a useful test result were included in the final analysis. A biopsy was considered representative if a pathologist confirmed the diagnosis of breast malignancy.

Statistical analyses of ex vivo test outcomes were all two-sided and performed using IBM SPSS v25. Significance was calculated using a Fisher exact test for categorical data. *P* values of <0.05 were considered significant.

### Reporting summary

Further information on research design is available in the Nature Research Reporting Summary linked to this article.

### Supplementary information

Supplementary figures

Reporting Summary

## Data Availability

The datasets generated during and/or analyzed during the current study are available from the corresponding author upon reasonable request.
