# Supplementary material for: Proof-of-concept study linking ex vivo sensitivity testing to neoadjuvant anthracycline-based chemotherapy response in breast cancer patients
Source: NPJ Breast Cancer. 2023 Sep 30;9:80. doi: 10.1038/s41523-023-00583-6 (PMC10542784; doi:10.1038/s41523-023-00583-6)
Supplement: Supplementary file 1 — Supplementary figures [file 41523_2023_583_MOESM1_ESM.pdf]

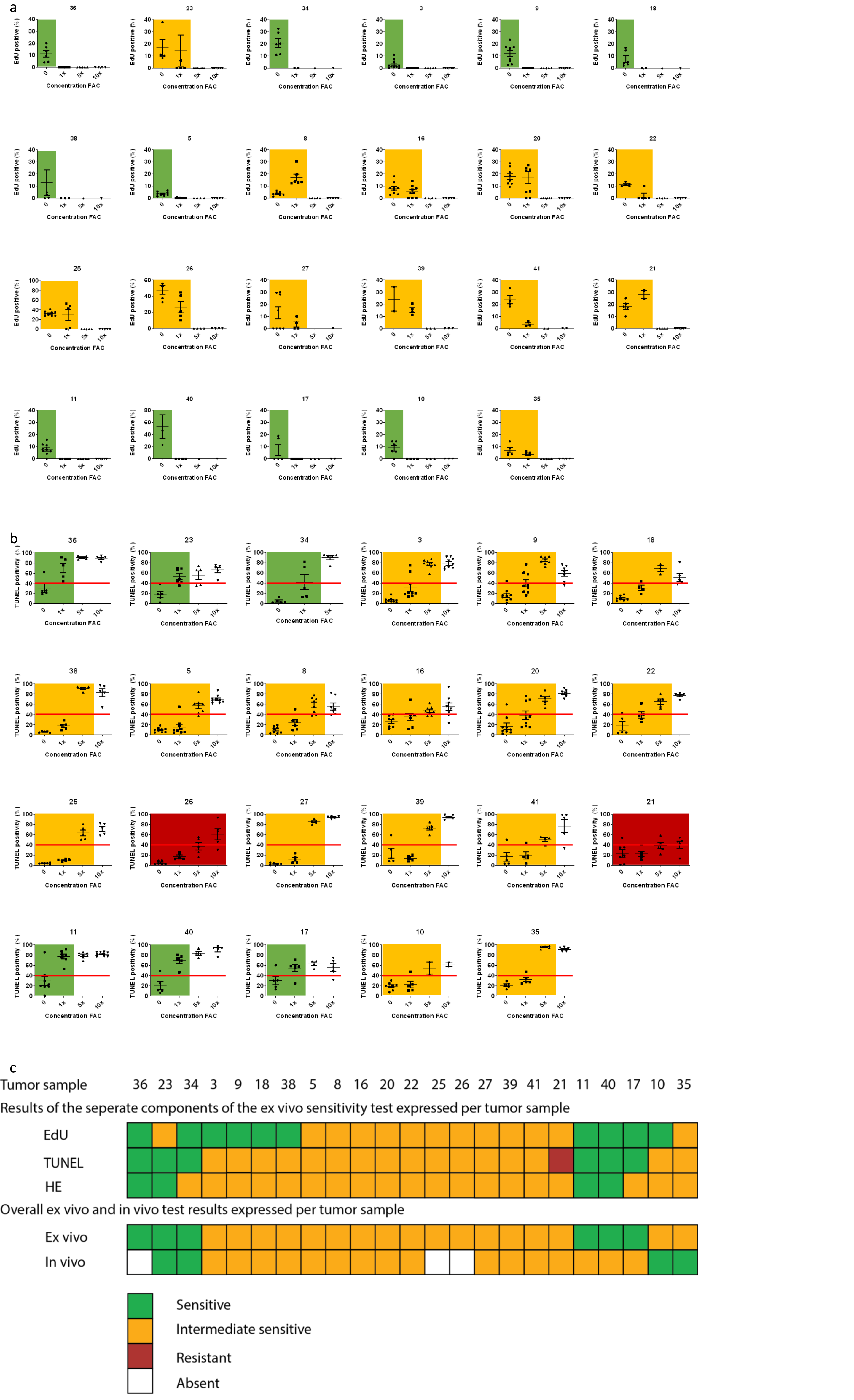

Supplementary Figure 1: **Sensitivity measured in individual biopsy samples.** a) Edu sensitive samples (green) have no proliferating cells in the lowest FAC concentration (1x). Intermediate sensitive samples (orange) have proliferating cells only in the lowest FAC concentration (1x), but not in concentrations 5x and 10x. Error bars are mean with standard error of the mean (SEM). b) Sensitive samples (green) have apoptotic cells (>40%, indicated by the red line) in the first FAC concentration (1x). Intermediate sensitive samples (orange) show apoptosis (>40%) in the highest concentration only (10x). Error bars are mean with standard error of the mean (SEM). c) Overview of all successful tests with the overall *ex vivo* sensitivity.

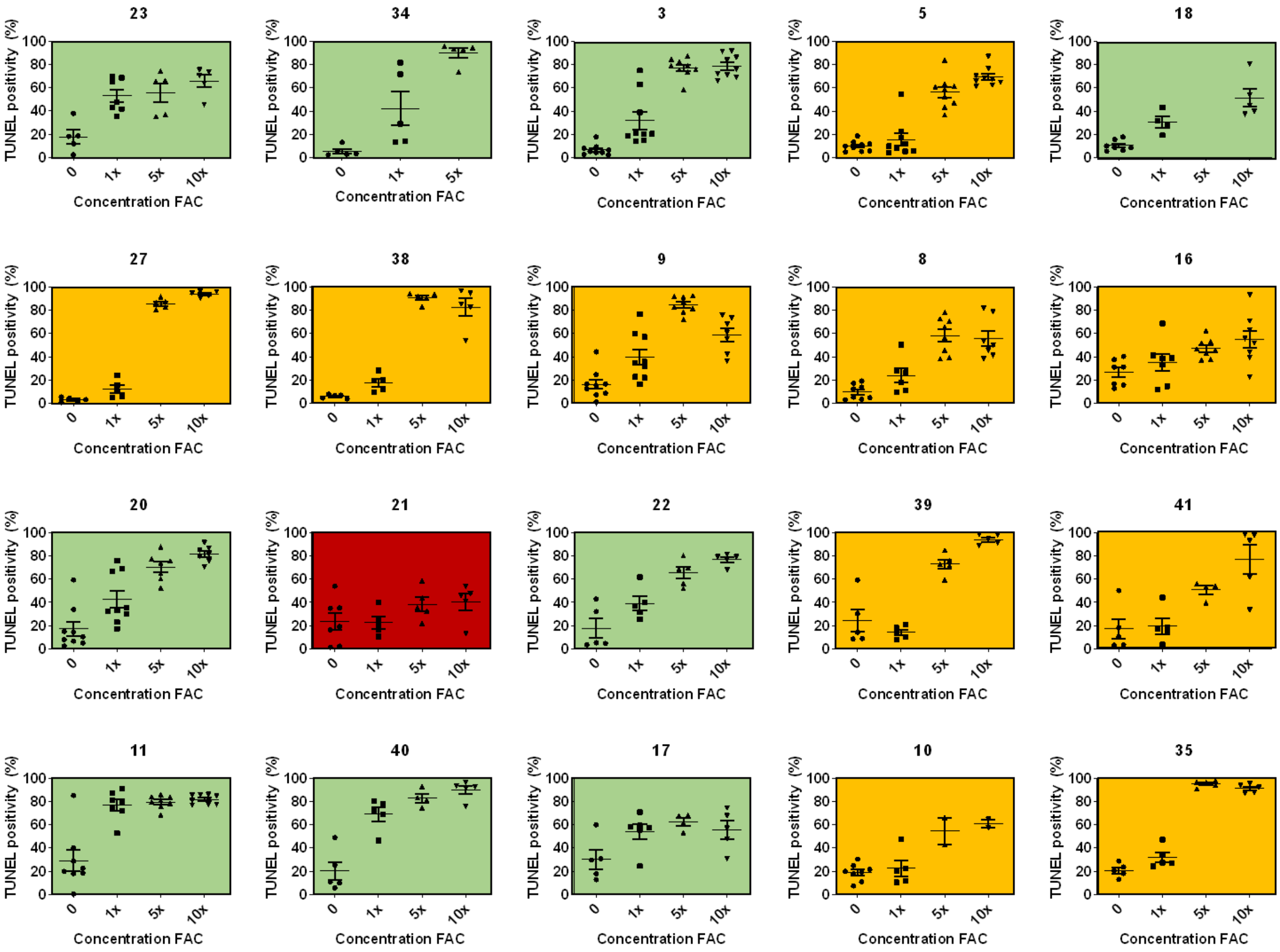

Supplementary Figure 2: Apoptosis (TUNEL) measured in biopsy samples, with alternative cut off. The alternative cut off was set to explore if this could predict *in vivo* outcome even better. Sensitive samples (green) have apoptotic cells (increase of >20%) in 1x SFC. Intermediate sensitive samples (orange) show apoptosis (increase of >20%) in 5x SFC. Resistant samples (red) show an increase of <20% in 5x SFC. Error bars are mean with standard error of the mean (SEM).
