# Supplementary material for: Proof-of-concept study linking ex vivo sensitivity testing to neoadjuvant anthracycline-based chemotherapy response in breast cancer patients
Source: NPJ Breast Cancer. 2023 Sep 30;9:80. doi: 10.1038/s41523-023-00583-6 (PMC10542784; doi:10.1038/s41523-023-00583-6)
Supplement: Supplementary file 2 — Reporting Summary [file 41523_2023_583_MOESM2_ESM.pdf]

nature portfolio

Corresponding author(s): A Jager

Last updated by author(s): 28-aug-2023

Reporting Summary

Nature Portfolio wishes to improve the reproducibility of the work that we publish. This form provides structure for consistency and transparency in reporting. For further information on Nature Portfolio policies, see our Editorial Policies and the Editorial Policy Checklist.

Statistics

For all statistical analyses, confirm that the following items are present in the figure legend, table legend, main text, or Methods section.

n/a	Confirmed	
☐	☒	The exact sample size (*n*) for each experimental group/condition, given as a discrete number and unit of measurement
☐	☒	A statement on whether measurements were taken from distinct samples or whether the same sample was measured repeatedly
☐	☒	The statistical test(s) used AND whether they are one- or two-sided<br>*Only common tests should be described solely by name; describe more complex techniques in the Methods section.*
☒	☐	A description of all covariates tested
☒	☐	A description of any assumptions or corrections, such as tests of normality and adjustment for multiple comparisons
☐	☒	A full description of the statistical parameters including central tendency (e.g. means) or other basic estimates (e.g. regression coefficient) AND variation (e.g. standard deviation) or associated estimates of uncertainty (e.g. confidence intervals)
☒	☐	For null hypothesis testing, the test statistic (e.g. F, t, r) with confidence intervals, effect sizes, degrees of freedom and P value noted<br>*Give P values as exact values whenever suitable.*
☒	☐	For Bayesian analysis, information on the choice of priors and Markov chain Monte Carlo settings
☒	☐	For hierarchical and complex designs, identification of the appropriate level for tests and full reporting of outcomes
☒	☐	Estimates of effect sizes (e.g. Cohen's *d*, Pearson's *r*), indicating how they were calculated

Our web collection on statistics for biologists contains articles on many of the points above.

Software and code

Policy information about availability of computer code

Data collection	Open clinica
Data analysis	IBM SPSS Statistics Data Editor version 28.0.1.0 (142)

For manuscripts utilizing custom algorithms or software that are central to the research but not yet described in published literature, software must be made available to editors and reviewers. We strongly encourage code deposition in a community repository (e.g. GitHub). See the Nature Portfolio guidelines for submitting code & software for further information.

Data

Policy information about availability of data

All manuscripts must include a data availability statement. This statement should provide the following information, where applicable:

- Accession codes, unique identifiers, or web links for publicly available datasets
- A description of any restrictions on data availability
- For clinical datasets or third party data, please ensure that the statement adheres to our policy

Data Availability
The datasets generated during and/or analysed during the current study are available from the corresponding author upon reasonable request.

Research involving human participants, their data, or biological material

Policy information about studies with human participants or human data. See also policy information about sex, gender (identity/presentation), and sexual orientation and race, ethnicity and racism.

Reporting on sex and gender	Both male and female were allowed to participated. Only female patients were included, due to the occurrence of BC.
Reporting on race, ethnicity, or other socially relevant groupings	For this study it is unlikely that differences in ethnicity or social background will influence the primary outcome of the study. Therefore, no predetermined requirements were set and all patients were allowed to participate, regardless of their ethnic or social background.
Population characteristics	Female patients with primary breast cancer.
Recruitment	All BC patients who had been scheduled to start with anthracycline-containing NAC without concomitant taxane treatment and had WHO performance status 0 or 1 without any psychologically interfering problem were potentially eligible and therefore recruited for the study.
Ethics oversight	This study was performed in line with the principles of the Declaration of Helsinki. Approval was granted by the Ethics Committee of the Erasmus University Rotterdam (MEC 16-600/ Netherlands Trial register NL5588).

Note that full information on the approval of the study protocol must also be provided in the manuscript.

Field-specific reporting

Please select the one below that is the best fit for your research. If you are not sure, read the appropriate sections before making your selection.

☒ Life sciences ☐ Behavioural & social sciences ☐ Ecological, evolutionary & environmental sciences

For a reference copy of the document with all sections, see nature.com/documents/nr-reporting-summary-flat.pdf

Life sciences study design

All studies must disclose on these points even when the disclosure is negative.

Sample size	For this pilot, we aimed to find a concordance between the ex vivo and in vivo response of at least 55% (P1), which was considered sufficiently promising to warrant further research. If concordance was less than 25% (P0) the ex vivo assay in its current format has no advantage over patient selection according to present standards. For the sample size calculation, the optimal Simon 2-stage design was applied 13. With an α and β of both 0.10, the required number of patients with a successful ex vivo test result to be included was 20. Of these 20 patients, 55% needed to be concordant to draw a final conclusion.
Data exclusions	No data was excluded.
Replication	NA
Randomization	Not relevant for this study.
Blinding	There was no blinding since all participants received the same handling.

Reporting for specific materials, systems and methods

We require information from authors about some types of materials, experimental systems and methods used in many studies. Here, indicate whether each material, system or method listed is relevant to your study. If you are not sure if a list item applies to your research, read the appropriate section before selecting a response.

Materials & experimental systems

n/a	Involved in the study	
☒	☐	Antibodies
☒	☐	Eukaryotic cell lines
☒	☐	Palaeontology and archaeology
☒	☐	Animals and other organisms
☐	☒	Clinical data
☒	☐	Dual use research of concern
☒	☐	Plants

Methods

n/a	Involved in the study	
☒	☐	ChIP-seq
☒	☐	Flow cytometry
☒	☐	MRI-based neuroimaging

Clinical data

Policy information about clinical studies

All manuscripts should comply with the ICMJE guidelines for publication of clinical research and a completed CONSORT checklist must be included with all submissions.

Clinical trial registration	NL5588
Study protocol	https://www.who.int/clinical-trials-registry-platform
Data collection	Patients were recruited and data was collected between November 2017 until March 2021
Outcomes	The primary outcome of our proof of concept study was the concordance between the ex-vivo sensitivity test and the in-vivo MRI response on anthracycline-based chemotherapy
